# Induction of Cyclooxygenase-2 by Overexpression of the Human NADPH Oxidase 5 (NOX5) Gene in Aortic Endothelial Cells

**DOI:** 10.3390/cells9030637

**Published:** 2020-03-06

**Authors:** Javier Marqués, Adriana Cortés, Álvaro Pejenaute, Eduardo Ansorena, Gloria Abizanda, Felipe Prósper, Juan José Martínez-Irujo, Carlos de Miguel, Guillermo Zalba

**Affiliations:** 1 Department of Biochemistry and Genetics, University of Navarra, 31008 Pamplona, Spain; jmarquesc@unav.es (J.M.); acortes.3@alumni.unav.es (A.C.); apejenaute@alumni.unav.es (Á.P.); eansorena@unav.es (E.A.); jjmirujo@unav.es (J.J.M.-I.); cdmiguel@unav.es (C.d.M.); 2 Navarra Institute for Health Research (IdiSNA), 31008 Pamplona, Spain; gabizanda@unav.es (G.A.); fprosper@unav.es (F.P.); 3 Hematology Service, Clínica Universidad de Navarra, University of Navarra, 31008 Pamplona, Spain; 4 CIBERONC, 31008 Madrid, Spain

**Keywords:** oxidative stress, NADPH oxidase 5, COX-2, PGE_2_, chronic infarction, PKC, NF-κB

## Abstract

Oxidative stress is a main molecular mechanism that underlies cardiovascular diseases. A close relationship between reactive oxygen species (ROS) derived from NADPH oxidase (NOX) activity and the prostaglandin (PG) biosynthesis pathway has been described. However, little information is available about the interaction between NOX5 homolog-derived ROS and the PG pathway in the cardiovascular context. Our main goal was to characterize NOX5-derived ROS effects in PG homeostasis and their potential relevance in cardiovascular pathologies. For that purpose, two experimental systems were employed: an adenoviral NOX5-β overexpression model in immortalized human aortic endothelial cells (TeloHAEC) and a chronic infarction in vivo model developed from a conditional endothelial NOX5 knock-in mouse. NOX5 increased cyclooxygenase-2 isoform (COX-2) expression and prostaglandin E_2_ (PGE_2_) production through nuclear factor kappa-light-chain-enhancer of activated B cells (NF-κB) in TeloHAEC. Protein kinase C (PKC) activation and intracellular calcium level (Ca^++^) mobilization increased ROS production and NOX5 overexpression, which promoted a COX-2/PGE_2_ response in vitro. In the chronic infarction model, mice encoding endothelial NOX5 enhanced the cardiac mRNA expression of COX-2 and PGES, suggesting a COX-2/PGE_2_ response to NOX5 presence in an ischemic situation. Our data support that NOX5-derived ROS may modulate the COX-2/PGE_2_ axis in endothelial cells, which might play a relevant role in the pathophysiology of heart infarction.

## 1. Introduction

Oxidative stress is considered a pivotal molecular mechanism underlying cardiovascular risk factors such as aging, obesity, insulin resistance, and tobacco. Oxidative stress is the result of an excessive accumulation of reactive oxygen species (ROS), which are physiological signaling molecules that, when overproduced, cause cellular damage [1]. One of the main sources of ROS is the NADPH oxidase (NOX) family, which consists of different enzymes specialized in the production of these molecules [2]. Seven different members make up the NOX family: five homologues (NOX1, NOX2, NOX3, NOX4, and NOX5) and two dual oxidases (DUOX1 and DUOX2). The main function of these oxidases is to transform molecular oxygen into superoxide anion, leading to an increase in ROS production [3]. NOX1, NOX2, NOX4, and NOX5 are expressed in the vascular wall [4] and are related to different cardiovascular diseases (CVDs), such as atherosclerosis, hypertension, heart failure, or myocardial infarction (MI) [5].

The function and relevance of NOX5, the last described homolog, remains unclear. Furthermore, the evolutionary loss of this gene in the rodent genome has complicated its study. In humans, the *NOX5* gene is located on chromosome 15, and, to date, six alternative splicing isoforms are known: α, β, γ, δ, ε, and ζ. In the vascular wall, NOX5-α and β have been characterized in endothelial cells and vascular smooth muscle cells [6]. NOX counterparts share different structural characteristics, but NOX5 is the only isoform that has ejection fraction (EF)-hand motifs. These motifs regulate NOX5 activity by intracellular calcium levels (Ca^++^). At increased levels of this ion, NOX5 undergoes a conformational change, promoting its phosphorylation by different kinases, including protein kinase C (PKC), which activates the enzyme [7,8].

Oxidative stress increases prostaglandin (PG) synthesis in different cell types by several regulatory pathways [9,10,11]. Altered PG production constitutes a key inflammatory response involved in cardiovascular pathologies, such as atherosclerosis [12]. PGs are potent biologically-active lipid molecules derived from arachidonic acid by the action of cyclooxygenase activity and different prostaglandin synthases. Cyclooxygenase-2 isoform (COX-2) is the main regulatory point of this pathway and is considered a biomarker for acute coronary disease, showing relevant implications in CVDs [13].

Interestingly, a well-established relationship exists between ROS derived from NADPH oxidases and enhanced COX-2 expression and activity. This NOX/COX-2 axis has been widely described for NOX2 in different cells, including macrophages [14,15,16]. However, the NOX5-derived effect over COX-2 expression remains unknown and little information is available about this crosstalk, especially in the cardiovascular context. In an adenocarcinoma in vitro model, acidic stimulation induced prostaglandin E_2_ synthase (PGES) expression. This effect derived from the induction of COX-2 activity by NOX5-ε [17]. In addition, sphingosylphosphorylcholine induced NOX5 activation in human keratinocytes, which promoted COX-2 overexpression and PGE_2_ production [18]. With this background, the aim of the present investigation was to determine whether an increase in NOX5-β expression and activity could enhance COX-2 expression and modulate the PG signaling pathway in endothelial cells, and its potential impact on cardiovascular pathophysiology.

## 2. Materials and Methods

### 2.1. Cell Culture

The TeloHAEC cell line, a clonal line immortalized by stably expressing hTERT (from human telomerase), was used as an in vitro model. These cells were grown in an adherent culture, at 37 °C and 5% CO_2_. Vascular Cell Basal Medium with Endothelial Cell Growth kit-VEGF was used to maintain the cell line (2% fetal bovine serum). Both cells and medium were purchased from ATCC (American Type Culture Collection, Manassas, VA, USA). Additionally, 0.1 mg/mL antibiotics (penicillin and streptomycin) and antimycotics (gentamicin) were added to the medium (Sigma Aldrich, Saint Louis, MO, USA). To perform the different assays, cells were passaged to culture plaques the day before, obtaining 80–90% confluence.

Certain stimulators were used for different times: angiotensin II (Ang II), phorbol 12-myristate 13-acetate (PMA), and ionomycin (Io) (Sigma Aldrich, Saint Louis, MO, USA). Different inhibitors were used for different times and at different concentrations: PDTC (ammonium pyrrolidinedithiocarbamate, Sigma Aldrich) for NF-κB (nuclear factor kappa-light-chain-enhancer of activated B cells)**** inhibition and ML-090 (Cayman Chemical, Ann Arbor, MI, USA) for NOX5 inhibition.

### 2.2. Adenoviral Infection

Two adenovirus previously developed by our group were used in this project [19]. One of them codifies for the *Homo sapiens* sequence of the *NOX5-β* gene isoform, which is the most generally expressed isoform in the human endothelium, and the other one codifies for a variant of Green Fluorescent Protein (GFP), which is an innocuous protein that was used as the control. Both were generated at the Gene Therapy Laboratory at CIMA (Centro de Investigación Médica Aplicada, Pamplona, Spain). The standard dose of adenovirus stated was multiplicity of infection (MOI) 50, which means that the number of colony-forming units of adenovirus added to the medium was 50-times the cells seeded. These adenoviruses are non-replicative, which allowed us to maintain a permanent MOI. For the same purpose, during the 3 h of the infection process, a basal medium only containing Vascular Cell Basal Medium, 2% fetal bovine serum, antibiotics, and antimycotics was used, avoiding cell proliferation. After 3 h, this medium was replaced by complete medium.

### 2.3. Quantitative Real-Time PCR

RNA from TeloHAEC was extracted using Trizol (Thermo Fisher, Waltham, MA, USA) and standard protocols. To obtain RNA from the murine heart, samples were homogenized in Trizol and a standard protocol was followed. cDNA was obtained from 1 µg of RNA by reverse transcription with the SuperScript III system (Thermo Fisher, Rockford, IL, USA). mRNA expression was quantified using Master Mix iQ™ SYBR^®^ Green Supermix (BioRad, Hercules, CA, USA). A standard PCR protocol was followed: 15 min incubation at 95 °C (ensuring enzyme activation), and 40 cycles of 3 steps (20 s at 95 °C, 15 s of annealing at the specific temperature of each gene and 10 s of elongation at 72 °C). All reactions were performed in triplicate. Finally, the threshold cycle was calculated (C_T_) and normalized with an endogenous control gene (*GAPDH*). 2^-ΔΔCT^ (∆∆C_T_ = (C_T_ analysed gene - C_T_ control gene) - ΔC_T_ control gene mean) was calculated and data were normalized with the control. Primers used to the amplification are available in Table 1.

### 2.4. Western Blot

RIPA Buffer (150 mM NaCl, 1% NP-40, 50 mM Tris Ph = 8, 0.5% sodium deoxycholate and 0.1% SDS) was used for protein extraction. Furthermore, protease inhibitors (Roche, Basel, Switzerland) were added to avoid sample digestion. Finally, extracts were sonicated to complete membrane degradation. Samples were dissolved in loading buffer and separated for 75 min at 110 V. After electrophoresis, proteins were transferred to a nitrocellulose membrane at 0.35 A for 75 min. The membrane was blocked using Tris-Buffered saline with 5% milk for 1 h. Finally, the membrane was incubated overnight at 4 °C with primary antibodies and was revealed after 1 h of incubation at room temperature with secondary antibodies using the ECL Prime Western Blotting Detection Reagent (GE Healthcare Amersham, Chicago, Illinois) (Table 2).

### 2.5. Measurement of ROS Production

Amplex Red (Thermo Fisher) was used to measure H_2_O_2_ production in intact cells. “Amplex Red” molecule oxidation by horseradish peroxidase using H_2_O_2_ secreted by cells allowed quantification. Standard protocols were followed, and a microplate fluorescence reader (PolarStar, BMG Labtech, Ortenberg, Germany) was employed, using 544 nm excitation and 590 nm emission wavelengths.

Dihydroethidium (DHE, Thermo Fisher) was used to identify semi-quantitatively superoxide anion production in intact cells. Briefly, we seeded cells in 96-well format plates and infected with the GFP or NOX5 adenovirus, as previously described. In some experiments, cells were stimulated for 1 h with PMA+Io or Ang II. Then, cells were incubated with 100 µM DHE and images were obtained in a ZOE Fluorescent Cell Imager (Bio-Rad, Hercules, CA, USA).

### 2.6. Quantification of Apoptosis

Cellular apoptosis was quantified in intact cells using the Caspase-Glo 3/7 assay kit (Promega, Madison, WI, USA) in 96-well plates and luminescence was measured in a microplate luminometer (Luminoskan Ascent, Thermo Fisher, Rockford, IL, USA). This Kit quantifies caspase-3 and caspase-7 activity by luminescence derived from the reaction.

### 2.7. Transient Transfection

Transfection was performed using Opti-MEM^TM^ medium (Gibco^TM^, Carlsbad, CA, USA) and the Lipofectamine 3000 Transfection Kit from Invitrogen (Carlsbad, CA, USA) for 20000 cells (TeloHAEC) in a 96-well format.

COX-2 promoter activity was measured with pDRIVE5-Lucia-hCOX2 (InvivoGen, Waltham, MA, USA) controlling the expression of a secreted luciferase enzyme and pSELECT-zeo-SEAP (InvivoGen) as a control plasmid to normalize the transfection efficiency. Both plasmids were used at a 1:1 proportion, and their activity was measured using QUANTI-LucGoldTM (InvivoGen) and QUANTI-Blue (InvivoGen) kits, respectively.

### 2.8. PG Quantification (ELISA)

Supernatant from 500,000 seeded cells in 6-well format plaques was collected and centrifuged at 525× *g* for 5 min at 4 °C, and precipitate was discarded. Samples were stored at −80 °C until use. Finally, supernatants were diluted 1:5 in phosphate buffer saline, and PGE_2_ levels were measured using an ELISA kit (Abcam^®^, Cambridge, United Kingdom).

### 2.9. MI Model and Echocardiography

Conditional knock-in mice for the *NOX5-β* gene were used in this study (NOX5^+/−^CRE^+/−^) (Figure S1). C57BL/6 mice only expressed NOX5 in endothelial cells after induction with tamoxifen (40 mg/kg) for 3 non-consecutive days, inducing endothelial-specific CRE recombinase activation. As a control group, we used endothelial cell-specific CRE recombinase expressing mice (CRE^+/−^). MI was induced in adult mice (15-week-old, male, *n* = 16/group) by permanent ligation of the left anterior descending coronary artery (LAD) with Prolene 7/0 (W8702, ETHICON), a nylon surgical non-resorbable suture, as previously described [20]. After surgery, mice recovery was evaluated, and weight was recorded every two days. Experiments were performed in accordance with European Communities Council Directives (2010/63/EU) guidelines for the care and use of laboratory animals and were approved by the University of Navarra Animal Research Review Committee (Protocol 106-17).

Transthoracic echocardiography was performed using the Vevo 770 high-resolution ultrasound system (Visual Sonics Inc., Toronto, Canada). The chest was shaved and mice were anesthetized with 2–3% isoflurane (IsoVet, B. Braun VetCare S.A., Tuttinglen, Germany), which was maintained until the procedure was finished. Mice were placed on a heating pad to maintain corporal heat and the heart rate was monitored during the procedure. Three measurements were performed until animals were sacrificed, 3 days before surgery (basal echocardiography), 2 and 28 days after ligation of the left anterior descending coronary artery. Echocardiography parameters were obtained as previously described [21,22]. The echocardiography parameters measured were the interventricular septum (IVS), left ventricular internal diameter (LVID), left ventricular posterior wall (LVPW), left ventricle volume (LV Vol), ejection fraction (EF), fractional shortening (FS), and left ventricle mass (LV Mass).

### 2.10. Histological Staining by Sirius Red

Tissue samples included in paraffin were cut into serial sections to create slices with a thickness of 3 µm. After rehydration, Sirius Red staining was performed by incubating samples with the solution for 30 min. Then, samples were again dehydrated and mounted with DPX (Panreac AppliChem^®^, Darmstadt, Germany). Images were obtained with a Nikon SMZ18 stereomicroscope and Nikon acquisition software NIS-Elements D 4.30.00 version (Nikon^®^, Minato, Japan).

### 2.11. Statistical Analysis

In vitro results were expressed as the mean ± standard error of the mean (SEM). Group normality was studied and then a t-test analysis was used to compare each group with the control group (considering the normality test results). In vivo results were also expressed as the mean ± SEM. Comparisons among three or more groups were analysed by an ANOVA test. To analyse the data distribution, the Shapiro–Wilk test was used, which justified the use of a parametric test. The analysis performed for echocardiographic results was done in a time-independent manner, with data only being compared by genotype. The statistical analysis was performed using GraphPad Prism 8 (GraphPad^®^, San Diego, CA, USA). Statistical significance was established as *p* < 0.05.

## 3. Results

### 3.1. In Vitro Model Characterization

NOX5 expression was analyzed in the TeloHAEC cell line at the baseline. NOX5 mRNA levels were scarce and no protein was detected by Western Blot (data not shown). These results show that NOX5 expression in this cell line is very low.

**Figure 1:**
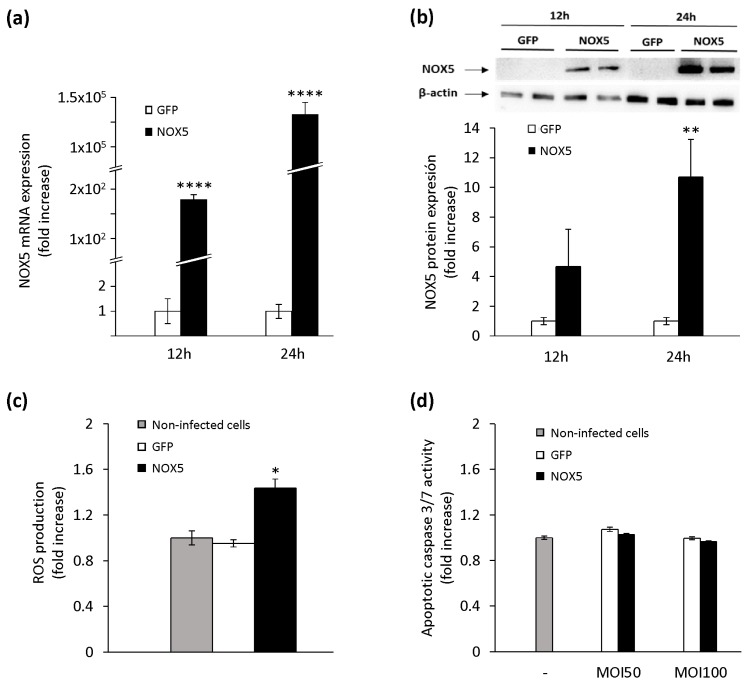
Characterization of NADPH oxidase (NOX)5-β overexpression in TeloHAEC cells infected with NOX5-β and control Green Fluorescent Protein (GFP) adenoviruses. (**a**) NOX5 mRNA levels after 12 and 24 h of infection. *n* = 6. **** *p* < 0.0001 vs. GFP. (**b**) Representative NOX5 and β-actin immunoblots of cells after 12 and 24 h of infection, and quantification of the NOX5 protein. *n* = 4. ** *p* < 0.01 vs. GFP. (**c**) ROS production of non-infected cells and cells infected with GFP or NOX5-β adenoviruses. *n* = 6. * *p* < 0.05 vs. non-infected and GFP-infected cells. (**d**) Apoptotic levels of non-infected cells and cells infected with GFP or NOX5-β adenoviruses at different multiplicity of infections (MOIs). *n* = 6.

TeloHAEC infection with NOX5-β adenovirus resulted in higher mRNA expression of this oxidase (Figure 1a). After 12 h of infection, NOX5 was already overexpressed, and this effect was much more intense after 24 h. NOX5 overexpression had no effect on the mRNA levels of other NOX homologs (Figure S2).

These data correlated with an enhanced NOX5 protein expression (Figure 1b). To test if this recombinant protein was functional, H_2_O_2_ production was measured after 24 h. The levels of H_2_O_2_ were higher in NOX5-infected cells than in GFP-infected or non-infected cells (Figure 1c), confirming that the recombinant protein exhibited enzymatic activity. Similarly, NOX5-infected cells exhibited higher superoxide generation than that observed in GFP-infected cells (Figure S3). Finally, we studied the effect of ROS overproduction on cell viability after adenoviral infection. No differences were found in apoptosis (caspase 3/7 activity) among non-infected cells and cells infected with GFP or NOX5-β adenoviruses, both at low (50) or high (100) MOIs (Figure 1d). All these data show that our in vitro model generates a functional NOX5 protein, which does not affect the cell viability.

### 3.2. Recombinant NOX5-β Activity Enhanced PGE_2_ Production via COX-2 Upregulation

**Figure 2:**
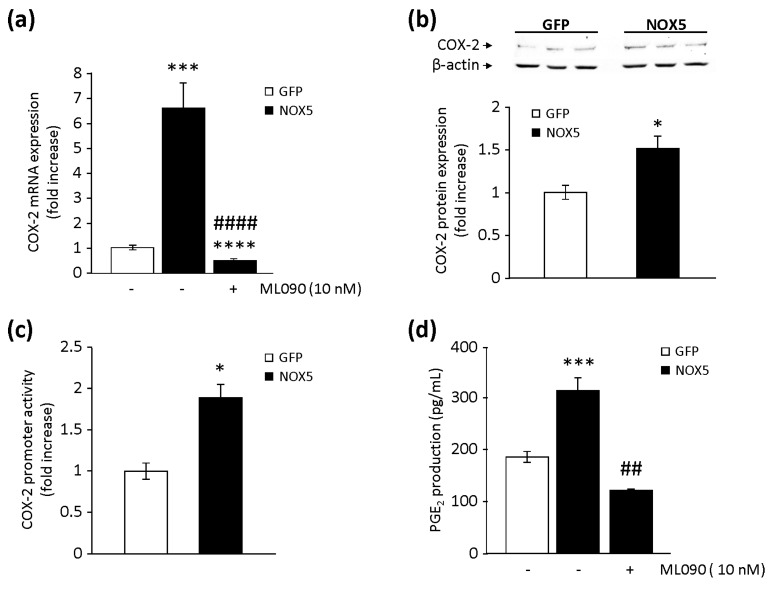
Effects of NOX5-β overexpression on prostaglandin (PG) biosynthesis in TeloHAEC. (**a**) COX-2 mRNA levels in NOX5-β- and GFP-infected cells for 12 h, in the presence or absence of ML090. *n* = 6. *** *p* < 0.001 vs. GFP, **** *p* < 0.0001 vs. GFP, and ^####^ *p* < 0.0001 vs. NOX5. (**b**) Representative COX-2 and β-actin immunoblots from cells infected for 12 h, and quantification of the COX-2 protein. *n* = 5. * *p* < 0.05 vs. GFP. (**c**) COX-2 promoter transcriptional activity of cells infected for 24 h. *n* = 6. * *p* < 0.05 vs. GFP. (**d**) PGE_2_ levels present in cell supernatant after 12 h of infection in the presence or absence of ML090. *n* = 6. *** *p* < 0.001 vs. GFP, and ^##^ *p* < 0.01 vs. NOX5.

A crosstalk between NOX5-derived ROS and COX-2 expression has been described in human keratinocytes [17]. Therefore, our next purpose was to study if this crosstalk was relevant in endothelial cells. After 12 h of infection, COX-2 mRNA levels were increased 6-fold in NOX5-β-infected cells compared with controls (Figure 2a). This effect was prevented by the specific inhibition of NOX5 with ML090 [23]. Similarly, higher levels of COX-2 protein were also detectable after 12 h of infection with NOX5-β adenovirus (Figure 2b). Then, we analysed if NOX5-mediated COX-2 mRNA upregulation was due to modulation of its transcriptional activity. First, cells transfected with a full human COX-2 promoter construct showed higher transcriptional activity when exposed to H_2_O_2_ (Figure S4). In addition, cells transfected with this construct and infected with NOX5-β adenovirus increased their transcriptional activity compared with control conditions (Figure 2c). Other genes involved in the PG biosynthesis pathway were analysed at mRNA levels. We found no differences between cells infected with NOX5-β or GFP for cytosolic phospholipase A_2_ (cPLA-2), cyclooxygenase-1 (COX-1), PGES, and PGI_2_ synthase (PTGIS) (data not shown).

To better address NOX5-derived ROS effects in PG homeostasis, PGE_2_ levels were measured. It has been demonstrated that NOX5 activation increases PGE_2_ production [17,18]. The levels of PGE_2_ were significantly higher in supernatants from NOX5-infected cells than in control cells. Specific inhibition of NOX5 oxidase activity completely prevented enhanced PGE_2_ production (Figure 2d).

### 3.3. PKC Stimulation Enhances NOX5-Mediated COX-2 Activation

**Figure 3:**
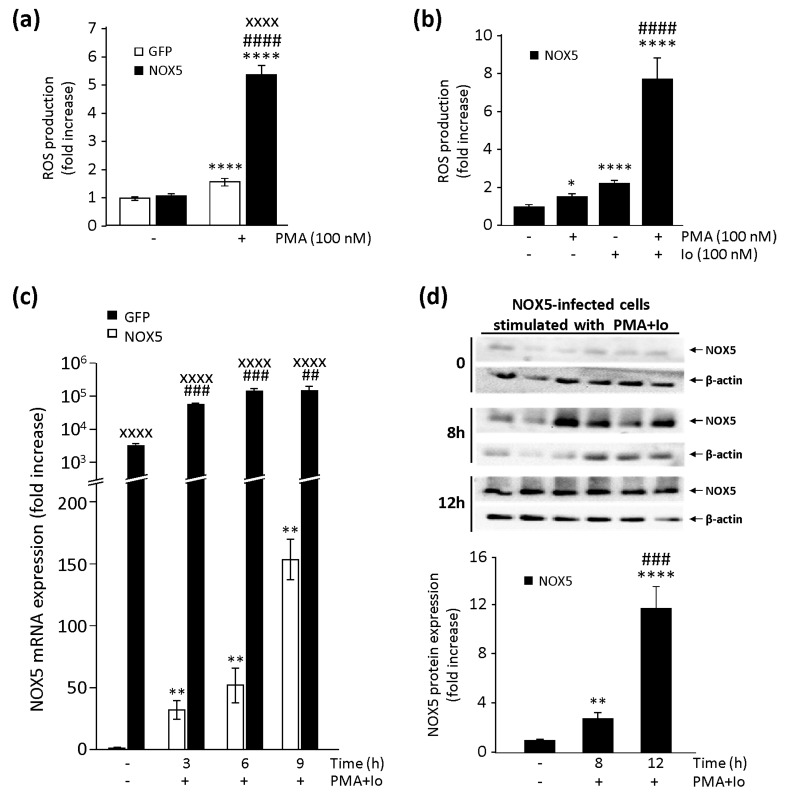
Characterization of NOX5 pharmacological stimuli in infected TeloHAEC. (**a**) ROS production measurements in NOX5-β- and GFP-infected cells for 24 h, and then stimulated with phorbol 12-myristate 13-acetate (PMA). *n* = 6. **** *p* < 0.0001 vs. non-stimulated GFP, ^####^ *p* < 0.0001 vs. non-stimulated NOX5, and ^xxxx^ *p* < 0.0001 vs. GFP+PMA. (**b**) ROS production measurements in NOX5-β-infected cells for 24 h, and then stimulated with PMA, ionomycin (Io), or a combination of both. *n* = 6. * *p* < 0.05 vs. non-stimulated NOX5, **** *p* < 0.0001 vs. non-stimulated NOX5, and ^####^ *p* < 0.0001 vs. NOX5+PMA or NOX5+Io. (**c**) NOX5 mRNA levels in NOX5-β- and GFP-infected cells for 24 h, and then stimulated with PMA+Io at different times. *n* = 6. ** *p* < 0.01 vs. non-stimulated GFP, ^##^ *p* < 0.01 vs. non-stimulated NOX5, ^###^ *p* < 0.001 vs. non-stimulated NOX5, and ^xxxx^ *p* < 0.0001 vs. the pertinent time of GFP-infected cells. (**d**) Representative NOX5 and β-actin immunoblots from cells infected for 24 h with NOX5-β adenovirus and stimulated with PMA+Io at different times, and quantification of NOX5 protein. *n* = 6. ** *p* < 0.01 vs. non-stimulated NOX5, **** *p* < 0.0001 vs. non-stimulated NOX5, and ^###^ *p* < 0.001 vs. 8 h-stimulated NOX5.

Given that NOX5 full activation involves numerous complex mechanisms, we promoted the activation of our in vitro model with the help of two pharmacological stimuli: PMA (a PKC activator) and Io (a Ca^++^ ionophore). PMA stimulation increased ROS production in both NOX5-β- and GFP-infected cells (Figure 3a), with this enhancement being much more notable in NOX5-β-infected cells. When NOX5-β-infected cells were treated with PMA in combination with Io, a synergic effect appeared in ROS production (Figure 3b and Figure S5).

Since PMA also enhanced ROS production in GFP-infected cells (Figure 3a), we wondered if the combination of PMA and Io stimuli could promote upregulation of the endogenous NOX5 gene in TeloHAEC cells. A combination of both stimuli increased NOX5 expression (Figure 3c). This stimulus produced an overexpression on NOX5 mRNA levels prolonged over time, reaching 150 times the basal levels in GFP-infected cells and 200,000 times those in NOX5-β-infected cells after 9 h of stimulation (Figure 3c). In NOX5-β-infected cells, maximum mRNA levels were reached 6 h after stimulation and were maintained over time. These effects at the mRNA level correlated with those at the protein level, at least in stimulated NOX5-β-infected cells (Figure 3d). Twelve hours after stimulation with a combination of both stimuli, we detected a 12-fold increase in NOX5 protein expression. We did not detect NOX5 protein in stimulated GFP-infected cells, probably due to the low levels of the endogenous oxidase in these cells (data not shown).

**Figure 4:**
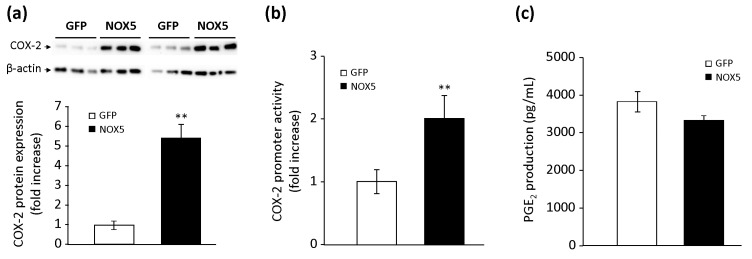
Effects of NOX5 pharmacological stimulation on PG biosynthesis. (**a**) Representative COX-2 and β-actin immunoblots of TeloHAEC infected for 24 h with GFP or NOX5-β adenovirus and stimulated with PMA+Io for 12 h, and quantification of COX-2 protein. *n* = 6. ** *p* < 0.01 vs. GFP. (**b**) COX-2 promoter transcriptional activity of TeloHAEC infected for 24 h with GFP or NOX5-β and stimulated with PMA+Io. *n* = 6. ** *p* < 0.01 vs. GFP. (**c**) PGE_2_ levels present in cell supernatant after 24 h of infection and stimulation with PMA+Io. *n* = 6.

Then, we analyzed the effect of these combined stimuli (PMA+Io) on the PG pathway. Both NOX5-β- and GFP-infected cells, when stimulated with the combination of PMA and Io, increased the COX-2 protein expression compared to non-stimulated infected-cells (data not shown). However, cells infected with NOX5-β and stimulated presented protein levels almost six times higher than stimulated GFP-infected cells (Figure 4a). Similarly, in cells transfected with the human COX-2 promoter construct, NOX5-β-infected cells and stimulated with the combined stimulus exhibited transcriptional activity two times higher than stimulated GFP-infected cells (Figure 4b). Finally, we found that the combination of PMA and Io increased the secretion of PGE_2_ 10-fold, both in NOX5-β- and GFP-infected cells, compared with non-stimulated infected cells (data not shown). However, we found no differences between stimulated NOX5-β- and GFP-infected cells (Figure 4c).

**Figure 5:**
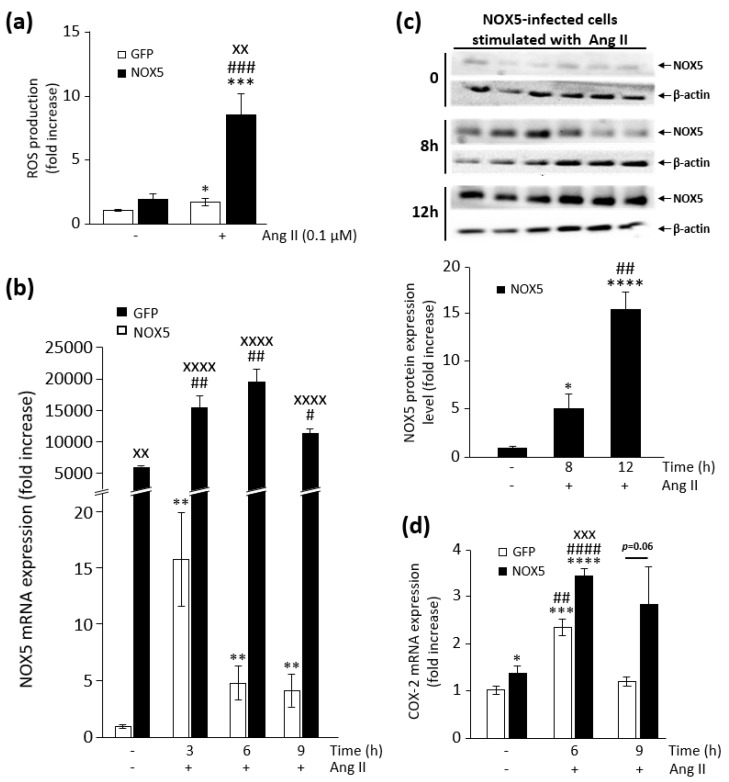
Characterization of the effect of NOX5 stimulation by angiotensin II (Ang II) in TeloHAEC. (**a**) ROS production measurements in NOX5-β- and GFP-infected cells for 24 h, and then stimulated with 0.1 μM Ang II. *n* = 6. * *p* < 0.05 vs. GFP, *** *p* < 0.001 vs. GFP, ^###^ *p* < 0.001 vs. NOX5, and ^xx^ *p* < 0.01 vs. GFP+Ang II. (**b**) NOX5 mRNA levels in NOX5-β- and GFP-infected cells for 24 h, and then stimulated with Ang II at different times. *n* = 6. ** *p* < 0.01 vs. non-stimulated GFP, ^#^ *p* < 0.05 vs. non-stimulated NOX5, ^##^ *p* < 0.01 vs. non-stimulated NOX5, and ^xx^ *p* < 0.01 and ^xxxx^ *p* < 0.0001 vs. the pertinent time of GFP-infected cells. (**c**) Representative NOX5 and β-actin immunoblots from cells infected for 24 h with NOX5-β adenovirus and stimulated with Ang II at different times, and quantification of NOX5 protein. *n* = 6. * *p* < 0.05 vs. non-stimulated NOX5, **** *p* < 0.0001 vs. non-stimulated NOX5, and ^##^ *p* < 0.01 vs. 8 h-stimulated NOX5. (**d**) COX-2 mRNA levels in NOX5-β- and GFP-infected cells for 24 h, and then stimulated with Ang II at different times. *n* = 6. * *p* < 0.05 vs. non-stimulated GFP, *** *p* < 0.001 vs. non-stimulated GFP, **** *p* < 0.0001 vs. non-stimulated GFP, ^##^ *p* < 0.01 vs. non-stimulated NOX5, ^####^ *p* < 0.001 vs. non-stimulated NOX5, and ^xxx^ *p* < 0.001 vs. 6 h-stimulated GFP.

Ang II, a physiological stimulus that exerts its effects through PKC, is able to activate NOX5 in endothelial cells [24]. As expected, Ang II stimulation increased ROS production in both NOX5-β- and GFP-infected cells (Figure 5a and Figure S5), with this enhancement being much more notable in NOX5-β-infected cells. Ang II also increased NOX5 mRNA levels in both GFP- and NOX5-infected cells (Figure 5b). This stimulation seems to have a punctual impact on cells infected with GFP adenovirus, while in NOX5-infected cells, this effect lasted longer. This effect had its correlation at the protein level in stimulated NOX5-β-infected cells (Figure 5c). Twelve hours after stimulation, we detected a 16-fold increase in NOX5 protein. We did not detect NOX5 protein in stimulated GFP-infected cells, probably due to the low levels of the endogenous oxidase in these cells (data not shown). Finally, both NOX5-β- and GFP-infected cells, when stimulated with Ang II, increased COX-2 mRNA expression compared to non-stimulated infected-cells (Figure 5d). However, cells infected with NOX5-β adenovirus presented higher COX-2 upregulation than stimulated GFP-infected cells.

### 3.4. NOX5 Increases NF-κB-Mediated COX-2 Expression

**Figure 6:**
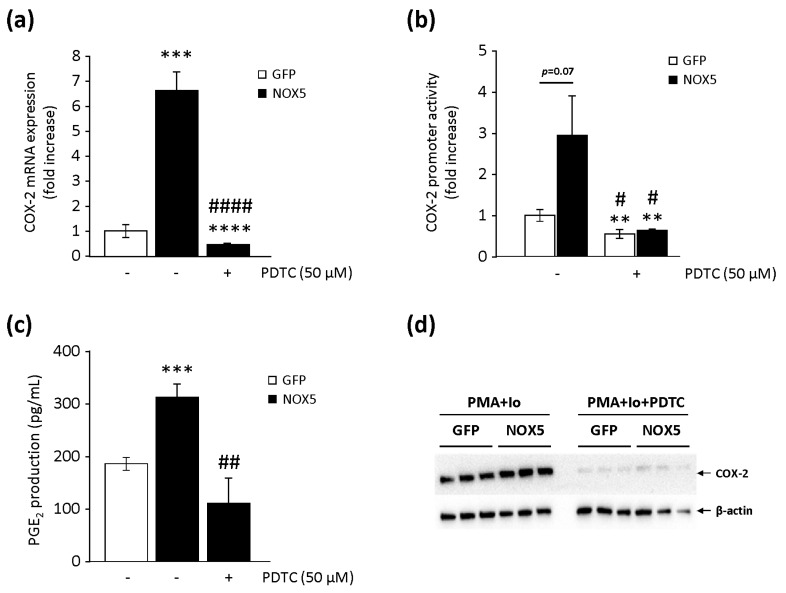
Role of nuclear factor kappa-light-chain-enhancer of activated B cells (NF-κB) in the NOX5/COX-2 axis in TeloHAEC. (**a**) COX-2 mRNA levels of NOX5-β- and GFP-infected cells for 12 h in the absence or presence of 50 µM ammonium pyrrolidinedithiocarbamate (PDTC). *n* = 6. *** *p* < 0.001 vs. non-inhibited GFP, **** *p* < 0.0001 vs. non-inhibited GFP, and ^####^ *p* < 0.0001 vs. non-inhibited NOX5. (**b**) COX-2 promoter transcriptional activity of NOX5-β- and GFP-infected cells for 24 h in the absence or presence of 50 µM PDTC. *n* = 6. ** *p* < 0.01 vs. non-inhibited GFP, and ^#^ *p* < 0.05 vs. non-inhibited NOX5. (**c**) PGE_2_ levels present in cell supernatants from NOX5-β- and GFP-infected cells for 12 h in the absence or presence of 50 µM PDTC. *n* = 6. *** *p* < 0.001 vs. non-inhibited GFP, and ^##^ *p* < 0.01 vs. non-inhibited NOX5. (**d**) Representative COX-2 and β-actin immunoblots from cells infected for 24 h, and stimulated with PMA+Io in the absence or presence of 50 µM PDTC. *n* = 3.

NF-κB is a relevant transcription factor that participates in ROS-mediated COX-2 upregulation [15,16,17]. Therefore, we studied whether it could play a crucial role in NOX5-mediated COX-2 upregulation. As shown in Figure 6a, the specific blockage of NF-κB by the PDTC inhibitor completely prevented the upregulated COX-2 mRNA levels found in NOX5-β-infected cells. Similar results were found when studying COX-2 promoter transcriptional activity (Figure 6b). Cells infected with NOX5-β enhanced its transcriptional activity, which is an effect that disappeared in the presence of PDTC. GFP-infected cells also suffered from a reduction in transcriptional activity in the presence of PDTC. Finally, the blockage of NF-κB with PDTC also prevented the increased PGE_2_ levels found in NOX5-β-infected cells (Figure 6c). In an additional experiment, we found that the exposure of NOX5-β- and GFP-infected cells to the pharmacological stimulus (PMA+Io) dramatically enhanced COX-2 protein expression (Figure 6d). This effect was even more intense in NOX5-β-infected cells than in GFP cells. Interestingly, this effect was softened in both groups when NF-κB was inhibited, supporting a key role for this transcription factor in COX-2 upregulation induced by NOX5-dependent ROS production.

### 3.5. Characterization of the Conditional Humanized NOX5-β Knock-In Mice in a Model of MI

We studied the effect of human endothelial NOX5 on mice survival and remodelling after permanent LAD ligation. NOX5^+/−^CRE^+/−^ mice had a survival rate similar to CRE^+/−^ mice after permanent LAD ligation (Figure S6). Animals that survived the surgery and beyond the first days were followed up for 28 days to assess echocardiographic post-MI remodelling. No differences were found between control CRE^+/−^ and NOX5^+/−^CRE^+/−^ mice in echocardiographic parameters measured at three different times: pre-MI baseline, 2 days after MI, and 28 days after MI (Table 3). Myocardial fibrosis increased in both control CRE^+/−^ and NOX5^+/−^CRE^+/−^ mice, compared with non-infarcted mice (Figure S7). No differences between groups were observed in myocardial interstitial or perivascular fibrosis.

### 3.6. Conditional NOX5-β Knock-In Mice and the Cardiac PG Pathway in MI

**Figure 7:**
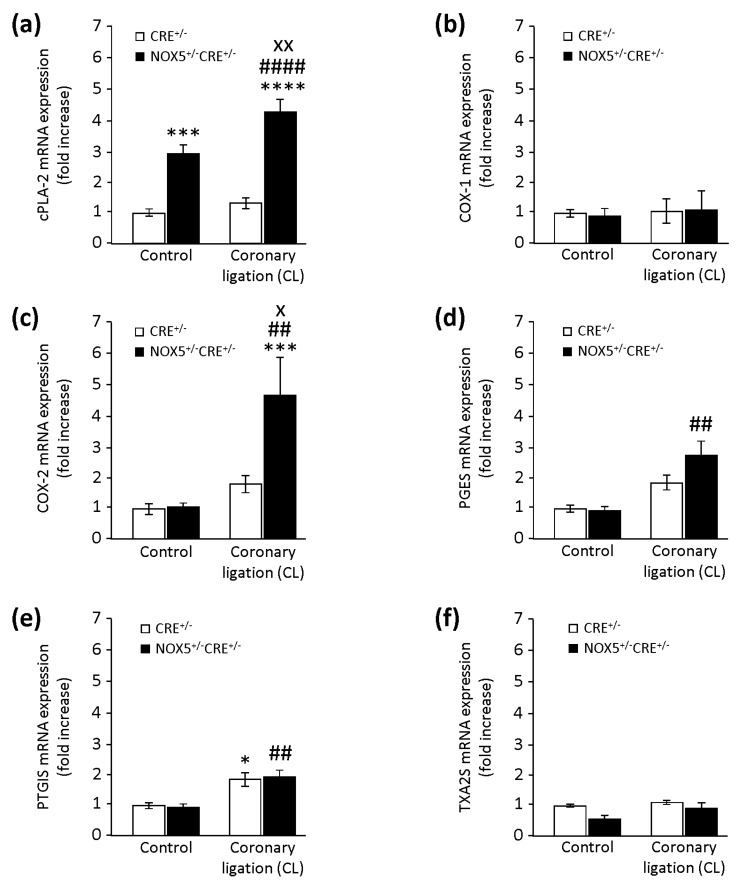
Quantification of enzymes involved in cardiac PG biosynthesis. (**a**) cPLA-2 mRNA expression in control and infarcted mice. *** *p* < 0.001 vs. CRE^+/−^, **** *p* < 0.0001 vs. CRE^+/−^, ^####^ *p* < 0.0001 vs. CRE^+/−^CL, and ^xx^ *p* < 0.01 vs. NOX5^+/−^CRE^+/−^. (**b**) COX-1 mRNA expression in control and infarcted mice. (**c**) COX-2 mRNA expression in control and infarcted mice. *** *p* < 0.001 vs. CRE^+/−^, ^##^ *p* < 0.01 vs. CRE^+/−^CL, and ^x^ *p* < 0.05 vs. NOX5^+/−^CRE^+/−^. (**d**) PGES mRNA expression in control and infarcted mice. ^##^ *p* < 0.01 vs. NOX5^+/−^CRE^+/−^. (**e**) PGI_2_ synthase (PTGIS) mRNA expression in control and infarcted mice. * *p* < 0.05 vs. CRE^+/−^, and ^##^ *p* < 0.01 vs. NOX5^+/−^CRE^+/−^. (**f**) Thromboxane A2 synthase (TXA2S) mRNA expression in control and infarcted mice. Control, control mice with no MI. Coronary ligation (CL), mice that suffered MI by LAD coronary ligation. CRE^+/−^, mice with the CRE^+/−^ genotype (*n* = 9 for CRE^+/−^ and NOX5^+/−^CRE^+/−^ groups). NOX5^+/−^CRE^+/−^ mice with the NOX5^+/−^CRE^+/−^ genotype (*n* = 12 for the CRE^+/−^CL group, and *n* = 13 for the NOX5^+/−^CRE^+/−^CL group).

We evaluated in vivo the effect of human endothelial NOX5 expression by analysing some enzymes involved in the cardiac PG pathway at the mRNA level (Figure 7). cPLA-2 mRNA was increased in NOX5^+/−^CRE^+/−^ mice compared with CRE^+/−^ mice at the baseline (Figure 7a). Besides, MI only increased cPLA-2 expression in NOX5^+/−^CRE^+/−^ mice (NOX5^+/−^CRE^+/−^CL). This might suggest that NOX5 enhances cPLA-2 levels, not only at the baseline, but also under ischemic conditions. COX-1 expression presented no differences between mice, neither at the baseline nor after MI (Figure 7b). Although we found no differences in COX-2 expression between groups at baseline conditions, MI promoted a significant upregulation in the NOX5^+/−^CRE^+/−^CL group compared with CRE^+/−^CL mice (Figure 7c). PGES expression presented no differences between mice at baseline conditions. Interestingly, MI only induced a significant increase in its expression in the NOX5^+/−^CRE^+/−^CL group (Figure 7d). Similarly, PTGIS expression presented no differences between mice at baseline conditions. In this case, MI increased its expression in both groups (Figure 7e). Finally, and although thromboxane A2 synthase (TXA2S) mRNA was reduced in the NOX5^+/−^CRE^+/−^ group compared with the CRE^+/−^ group at baseline conditions, this difference did not reach statistical significance (*p* = 0.07) (Figure 7f). MI did not exert any changes in its expression between groups.

Finally, we evaluated NOX2 and NOX4, whose expression presented no differences between mice at baseline conditions. MI increased their expression similarly in both groups of animals *(*Figure S8*)*.

## 4. Discussion

The main findings obtained in this study are the following: (i) NOX5-derived ROS lead to COX-2 upregulation and increased PGE_2_ production in TeloHAEC; (ii) NF-κB mediates the NOX5/COX-2/PGE_2_ axis in TeloHAEC; (iii) stimuli activating PKC and Ca^++^ mobilization dramatically enhances NOX5 expression and activity; and (iv) endothelial NOX5 overexpression alters the cardiac PG pathway in a chronic infarction mouse model. This work demonstrates, for the first time, a crosstalk between NOX5 and COX-2 in vascular endothelial cells, supporting a potential key role in CV pathophysiology.

PG biosynthesis may participate in CVDs, as atherosclerosis [25] or heart failure, by promoting inflammatory responses [26]. Oxidative stress seems to turn on this pathway, among other ways, by promoting COX-2 upregulation [10,11]. However, recent data has shown that, in some cases, ROS might also participate in anti-inflammatory responses. Aureusidin, a natural flavonoid, presented anti-inflammatory effects in LPS-stimulated RAW 264.7 macrophages by increasing cellular ROS levels [27]. Previous studies have demonstrated the relevant involvement of other NOX isoforms, mainly NOX2, in COX-2 activation [14,15,16]. Nevertheless, there is little information about the relationship between NOX5 and COX-2, only in adenocarcinoma cells [17] and keratinocytes [18], with no information in vascular wall cells. Our in vitro studies demonstrate that NOX5 overexpression induces an increased production of ROS, which promotes COX-2 upregulation in human endothelial cells. Accordingly, ML090, a specific NOX5 inhibitor, completely prevented this effect. Furthermore, stimulation with H_2_O_2_ of COX-2 promoter-transfected cells increased its transcriptional activity, thus demonstrating a direct involvement of ROS in COX-2 upregulation. Additional studies have shown that activation of the NOX-ROS axis translates into enhanced PGE_2_ production. Urban particulate matter stimulates NOX activity, which leads to an enhanced COX-2 expression and PGE_2_ production in human fibroblast-like synoviocytes [28]. Similarly, irradiation promotes an inflammatory response via NOX-derived ROS, which activates the COX-2/PGE_2_ pathway in RAW 264.7 macrophages [29]. As expected, NOX5-mediated COX-2 activation was associated with increased levels of PGE_2_ in endothelial cells, which is an effect that was abolished by specifically inhibiting NOX5. Collectively, our findings support a relevant role for the NOX5/COX-2/PGE_2_ pathway in inflammation.

PKC activation and Ca^++^ mobilization are two main mechanisms involved in NOX5 activation [7,8,19]. Previous studies have described that a combination of PMA and Io, which activates PKC and increases intracellular Ca^++^ levels, respectively, activated NOX5 in HEK 293 [30] and LX-2 [19] cell lines. As expected, the use of this pharmacological stimulus promoted the upregulation of endogenous and recombinant NOX5, and increased ROS production, both in NOX5-β- and GFP-infected cells. In addition, stimulation with PMA+Io increased COX-2 protein levels in both infected cells, with this effect being more noticeable in those infected with NOX5-β. Finally, the stimulus enhanced PGE_2_ secretion in both groups of cells, although no differences were found between them. These results support that the mechanisms of NOX5 and PGE_2_ in this experimental model may be divergent, though we cannot discard other relevant players that could also modulate the final levels of PGE_2_, including PGE_2_ synthase.

In the same way, Ang II is a hormone involved in the pathophysiology of hypertension and atherosclerosis, which also exerts its effects through PKC [31,32]. In fact, Ang II is an activator of NOX5 in human microvascular endothelial cells [24]. Similarly, as described above, we found that Ang II induced the upregulation of endogenous and recombinant NOX5 and increased ROS production, in both NOX5-β- and GFP-infected cells. Finally, the stimulus upregulated COX-2 expression in both infected cells, with this effect being longer in those infected with NOX5-β.

Some studies have linked COX-2 activation with ROS production and NF-κB in RAW 264.7 macrophages [33] and in vascular cells from hypertensive rats [34]. Interestingly, NF-κB is involved in NOX5-induced COX-2 activation in adenocarcinoma cells [17]. Nonetheless, there is no available information about signaling pathways involved in the NOX5/COX-2 axis in vascular endothelial cells. Our findings showing that incubation with PDTC inhibitor fully prevented COX-2 activation and PGE_2_ production in NOX5-infected endothelial cells demonstrated that NF-κB is the key regulator of these processes.

A relationship exists between the production of PGs and atherosclerosis. First, atorvastatin inhibited extracellular matrix metalloproteinase inducer via the COX-2/PGE_2_ pathway, indicating that this PG could play a role in atherosclerotic plaque instability in THP-1 macrophages [35]. Second, oxidized lipids of low-density lipoproteins increased the expression of COX-2 and membrane-bound PGES-1 in U937 macrophages, which might favor plaque instability. Oxidative stress was proposed to play a key role in this pathological pathway [36]. Therefore, our findings demonstrating that NOX5-mediated ROS production affects COX-2 activation and PGE_2_ production could have biological relevance in atherosclerotic pathophysiology. In agreement with this, Guzik et al. [37] demonstrated that NOX5 contributed to vascular oxidative stress in human coronary artery disease. As a matter of fact, NOX5 is expressed in the endothelium in early lesions and in vascular smooth muscle cells in advanced coronary lesions.

Likewise, oxidative stress is also related to MI, in which reperfusion of the affected zone leads to ROS overproduction. NOX1, NOX2, and NOX4 homologs are principal sources of these molecules in the heart [38], but knowledge of NOX5 in this case is scarce. Interestingly, NOX5 expression was increased in intramyocardial blood vessels and cardiomyocytes after acute MI in humans [39]. The authors speculate that the cardiac upregulation of NOX5 might play a key role in MI-induced remodelling. In this sense, our in vivo study reveals that NOX5 could play a role in PG homeostasis in the heart. At basal conditions, NOX5 upregulates cPLA-2 expression, which increases, even more, after chronic MI. Moreover, MI induces COX-2 and PGES upregulation in mice expressing NOX5. These data suggest that endothelial NOX5 expression might facilitate the turn on of the COX-2/PGES response, slightly in baseline situations and in a more accentuated manner under ischemic conditions. Although it has been traditionally accepted that COX-2 may be deleterious in cardiovascular pathophysiology, its role remains controversial, and recent studies support a protective role in this context [40]. On the one hand, different *COX-2* gene variants have been associated with different cardiovascular pathologies [41,42]. A murine model of cardiac-specific COX-2 overexpression, which presented higher PGE_2_ production, acquired protection to acute MI [43]. In addition, COX-2 specific inhibitors have presented severe secondary effects, including cardiovascular ones, which could be explained by the lack of a COX-2 protective role [44]. Therefore, future studies may be necessary to ascertain the real role of the NOX5/COX-2 axis in ischemic pathologies.

In summary, we demonstrated that NOX5-derived ROS upregulate COX-2 expression and PGE_2_ production in human endothelial cells via NF-κB. This NOX5/COX-2 pathway can be mediated via PKC activation. From a biomedical point of view, these results may be relevant, given that the COX-2/PGE2 pathway might also be activated by NOX5 in ischemic situations, and thus participate in cardiac remodelling after MI.

## Figures and Tables

**Table 1:** Primer sequences for quantitative real-time PCR analysis.

Gene	Forward Sequence	Reverse Sequence
*Hs cPLA-2*	GAAGATTCTCAGGTTTTAAAGACGC	GGCATCCATTAACGTAATCTCCAA
*Hs COX-1*	AAGTACCAGGTGCTGGATG	TGATGGTCTCCCCTATGA
*Hs COX-2*	TGCGGGAACACAACAGAGT	TAGCCACTCAAGTGTTGCAC
*Hs PGES*	AGGATGCCCTGAGACACGGAG	CCCAGGAAAAGGAAGGGGTAG
*Hs PTGIS*	ACATCTTTACTATACTGGTTGGGGG	TGTGGAGAAGAGTCAGTTTCATC
*Hs NOX1*	GCAGGGAGACAGGTGCCTTTTCC	CTACAGACTTGGGGTGGGAGGT
*Hs NOX2*	TTCCAGTGCGTGCTGCTCA	CTGCGGTCTGCCCACGTAC
*Hs NOX4*	CTGGCTCGCCAACGAAGGGG	GCTTGGAACCTTCTGTGATCCTCGG
*Hs NOX5*	ATGAGTGGCACCCCTTCACCATCAG	TCAGCAGGCTCACAAACCACTCGAA
*Hs GAPDH*	CCAAGGTCATCCATGACAAC	TGTCATACCAGGAAATGAGC
*Mm cPLA-2*	ACGTGATGTGCCGGTGG	AAGAGAGGCAAAGGACACCG
*Mm COX-1*	ACTCACAGTGCGGTCCAAC	AACTCCCTTCTCAGCAGCAG
*Mm COX-2*	TTCGGGAGCACAACAGAGT	TAACCGCTCAGGTGTTGCAC
*Mm PGES*	AGGATGCGCTGAAACGTGGAG	CCGAGGAAGAGGAAAGGATAG
*Mm PTGIS*	TTGTCAGCGGGGGATAAA	GACCCATATTCCCCTGTGTG
*Mm TXA2S*	AACAGAATGGCCTCAGGTCT	AGTTCACAGGCTTGGCTGAT
*Mm NOX2*	ACTCCTTGGGTCAGCACTGG	GTTCCTGTCCAGTTGTCTTCG
*Mm NOX4*	GGAGACTGGACAGAACGATTCC	TGTATAACTTAGGGTAATTTCTAGAGTGAATGA
*Mm GAPDH*	TGCTGAGTATGTCGTGGAGTCTA	CATTGCTGACAATCTTGAGTGAG

Hs, *Homo sapiens*; Mm, *Mus musculus*; c-PLA-2, cytosolic phospholipase A2; COX, cyclooxygenase; NOX, NADPH oxidase; PGES, prostaglandin E synthase; PTGIS, prostaglandin I2 synthase; GAPDH, glyceraldehyde-3-phosphate dehydrogenase; TXA2S, thromboxane A2 synthase.

**Table 2:** Primary and secondary antibodies.

Protein	Band Molecular Weight, kDa	Provider	Dilution
**Primary antibodies**			
Anti-NOX5 (polyclonal, rabbit)	75	Abcam	1:500
Anti-COX-2 (monoclonal, rabbit)	72	Cell Signalling	1:1000
Anti-β-actin (monoclonal, mouse)	42	Sigma	1:10000
**Secondary antibodies**			
Anti-rabbit (polyclonal, donkey)	-	GE Healthcare	1:10000
Anti-mouse (monoclonal, goat)	-	GE Healthcare	1:4000

**Table 3:** Echocardiographic parameters studied in left anterior descending coronary artery (LAD) coronary ligation mice at baseline and post-MI.

	Basal		2 Days		28 Days	
	NOX5^+/−^CRE^+/−^	CRE^+/−^	NOX5^+/−^CRE^+/−^	CRE^+/−^	NOX5^+/−^CRE^+/−^	CRE^+/−^
IVS;d (mm)	0.58 ± 0.01	0.56 ± 0.01	0.68 ± 0.03	0.63 ± 0.02	0.76 ± 0.02	0.73 ± 0.02
LVID;d (mm)	4.45 ± 0.09	4.50 ± 0.06	4.63 ± 0.08	4.69 ± 0.09	5.12 ± 0.14	5.41 ± 0.19
LVPW;d (mm)	0.56 ± 0.01	0.56 ± 0.01	0.62 ± 0.01	0.62 ± 0.03	0.71 ± 0.01	0.71 ± 0.02
IVS;s (mm)	0.70 ± 0.01	0.69 ± 0.01	0.79 ± 0.02	0.73 ± 0.02	0.87 ± 0.02	0.85 ± 0.02
LVID;s (mm)	3.22 ± 0.09	3.23 ± 0.06	3.82 ± 0.10	3.89 ± 0.09	4.15 ± 0.18	4.42 ± 0.22
LVPW;s (mm)	0.69 ± 0.01	0.69 ± 0.02	0.75 ± 0.01	0.74 ± 0.02	0.87 ± 0.03	0.84 ± 0.02
LV Vol;d (µL)	90.70 ± 4.18	92.51 ± 2.81	99.22 ± 4.17	101.26 ± 4.69	126.48 ± 8.14	136.45 ± 16.02
LV Vol;s (µL)	41.83 ± 2.90	43.23 ± 2.01	62.45 ± 3.94	65.47 ± 3.72	79.30 ± 8.43	87.02 ± 8.26
% EF	53.75 ± 1.50	54.18 ± 1.24	36.55 ± 1.73	35.39 ± 1.90	38.87 ± 2.99	37.34 ± 3.23
% FS	27.45 ± 1.20	29.59 ± 1.82	17.58 ± 0.94	16.98 ± 1.03	19.18 ± 1.66	18.44 ± 1.82
LV Mass (mg)	90.82 ± 3.18	90.45 ± 2.78	115.86 ± 6.32	110.91 ± 4.97	159.80 ± 7.84	172.80 ± 12.41
Corrected LV Mass (mg)	72.55 ± 2.53	71.99 ± 2.31	90.44 ± 5.41	89.95 ± 3.69	127.89 ± 6.28	138.24 ± 9.93

Data are represented as the mean ± SEM. CRE^+/−^, mice with the CRE genotype (*n* = 12); NOX5^+/−^CRE^+/−^, mice with the NOX5^+/−^CRE^+/−^ genotype (*n* = 13); IVS, interventricular septum; LVID, left ventricular internal diameter; LVPW, left ventricular posterior wall; LV Vol, left ventricle volume; EF, ejection fraction; FS, fractional shortening; LV Mass, left ventricle mass; d, diastole; s, systole.

## Supplementary Materials

The following are available online at https://www.mdpi.com/2073-4409/9/3/637/s1: Figure S1: Generation of the endothelial CRE-expressing knock-in mouse and experimental design; Figure S2: Effects of NOX5-β overexpression on NOX expression in TeloHAEC; Figure S3: Effect of NOX5-β overexpression on DHE oxidation in TeloHAEC; Figure S4: Effect of H_2_O_2_ on COX-2 promoter transcriptional activity in TeloHAEC; Figure S5: Effect of stimulation with PMA+Io or angiotensin II (Ang II) on superoxide production in GFP- and NOX5-β-infected TeloHAECs; Figure S6: Survival analysis after permanent ligation of the left anterior descending coronary artery; Figure S7: Myocardial interstitial and perivascular fibrosis; Figure S8: Quantification of NOX enzymes in the heart of mice.

## References

[1] Costantino S., Paneni F., Cosentino F. (2016). Ageing, metabolism and cardiovascular disease. J. Physiol..

[2] Takac I., Schröder K., Brandes R.P. (2012). The Nox family of NADPH oxidases: Friend or foe of the vascular system?. Curr. Hypertens. Rep..

[3] Thomsom M.J., Puntmann V., Kaski J.C. (2007). Atherosclerosis and oxidant stress: The end of the road for antioxidant vitamin treatment?. Cardiovasc. Drugs Ther..

[4] Touyz R.M., Briones A.M. (2011). Reactive oxygen species and vascular biology: Implications in human hypertension. Hypertens. Res..

[5] Zhang Y., Murugesan P., Huang K., Cai H. (2020). NADPH oxidases and oxidase crosstalk in cardiovascular diseases: Novel therapeutic targets. Nat. Rev. Cardiol..

[6] Pandey D., Patel A., Patel V., Chen F., Qian J., Wang Y., Barman S.A., Venema R.C., Stepp D.W., Rudic R.D. (2012). Expression and functional significance of NADPH oxidase 5 (Nox5) and its splice variants in human blood vessels. Am. J. Physiol. Heart Circ. Physiol..

[7] Chen F., Wang Y., Barman S., Fulton D.J. (2015). Enzymatic regulation and functional relevance of NOX5. Curr. Pharm. Des..

[8] Chen F., Yu Y., Haigh S., Johnson J., Lucas R., Stepp D.W., Fulton D.J. (2014). Regulation of NADPH oxidase 5 by protein kinase C isoforms. PLoS ONE.

[9] Yang C.M., Chen Y.W., Chi P.L., Lin C.C., Hsiao L.D. (2017). Resveratrol inhibits BK-induced COX-2 transcription by supressing acetylation of AP-1 and NF-ᴋB in human rheumatoid arthritis synovial fibroblasts. Biochem. Pharmacol..

[10] Zamamiri-Davis F., Lu Y., Thompson J.T., Prabhu K.S., Reddy P.V., Sordillo L.M., Reddy C.C. (2002). Nuclear factor-kappaB mediates over-expression of cyclooxygenase-2 during activation of RAW 264.7 macrophages in selenium deficiency. Free Radic. Biol. Med..

[11] Wei X., Zhang X., Flick L.M., Drissi H., Schwarz E.M., O’Keefe R.J. (2009). Titanium particles stimulate COX-2 expression in synovial fibroblasts through an oxidative stress-induced, calpain-dependent, NF-kappaB pathway. Am. J. Physiol. Cell Physiol..

[12] Hirsh P.D., Campbell W.B., Willerson J.T., Hillis L.D. (1981). Prostaglandins and ischemic heart disease. Am. J. Med..

[13] Gomez I., Foudi N., Longrois D., Norel X. (2013). The role of prostaglandin E2 in human vascular inflammation. Prostaglandins Leukot. Essent. Fatty Acids.

[14] Tsai M.H., Lin Z.C., Liang C.J., Yen F.L., Chiang Y.C., Lee C.W. (2014). Eupafolin inhibits PGE2 production and COX2 expression in LPS-stimulated human dermal fibroblasts by blocking JNK/AP-1 and Nox2/p47(phox) pathway. Toxicol. Appl. Pharmacol..

[15] Kim H.G., Kim Y.R., Park J.H., Khanal T., Choi J.H., Do M.T., Jin S.W., Han E.H., Chung Y.H., Jeong H.G. (2015). Endosulfan induces COX-2 expression via NADPH oxidase and the ROS, MAPK, and Akt pathways. Arch. Toxicol..

[16] Khanal T., Kim H.G., Do M.T., Choi J.H., Chung Y.C., Kim H.S., Park Y.J., Jeong T.C., Jeong H.G. (2014). Genipin induces cyclooxygenase-2 expression via NADPH oxidase, MAPKs, AP-1, and NF-ᴋB in RAW 264.7 cells. Food Chem. Toxicol..

[17] Zhou X., Li D., Resnick M.B., Wands J., Cao W. (2013). NADPH oxidase NOX5-S and nuclear factor ᴋB1 mediate acid-induced microsomal prostaglandin E synthase-1 expression in Barrett’s esophageal adenocarcinoma cells. Mol. Pharmacol..

[18] Choi H., Kim S., Kim H.J., Kim K.M., Lee C.H., Shin J.H., Noh M. (2010). Sphingosylphosphorylcholine down-regulates filaggrin gene transcription through NOX5-based NADPH oxidase and cyclooxygenase-2 in human keratinocytes. Biochem. Pharmacol..

[19] Andueza A., Garde N., García-Garzón A., Ansorena E., López-Zabalza M., Iraburu M.J., Zalba G., Martínez-Irujo J.J. (2018). NADPH oxidase 5 promotes proliferation and fibrosis in human hepatic stellate cells. Free Radic. Biol. Med..

[20] Pelacho B., Nakamura Y., Zhang J., Ross J., Heremans Y., Nelson-Holte M., Lemke B., Hagenbrock J., Jiang Y., Prosper F. (2007). Multipotent adult progenitor cell transplantation increases vascularity and improves left ventricular function after myocardial infarction. J. Tissue Eng. Regen. Med..

[21] Nakamura Y., Yoshiyama M., Omura T., Yoshida K., Kim S., Takeuchi K., Iwao H., Yoshikawa J. (2002). Transmitral inflow pattern assessed by Doppler echocardiography in angiotensin II type 1A receptor knock out mice with myocardial infarction. Circ. J..

[22] Benavides-Vallve C., Corbacho D., Iglesias-Garcia O., Pelacho B., Albiasu E., Castaño S., Muñoz-Barrutia A., Prosper F., Ortiz-de-Solozarno C. (2012). New strategies for echocardiographic evaluation of left ventricular function in a mouse model of long-term myocardial infarction. PLoS ONE.

[23] Dao V.T., Elbatreek M.H., Altenhöfer S., Casas A.I., Pachado M.P., Neullens C.T., Knaus U.G., Schmidt H.H.H.W. (2019). Isoform-selective NADPH oxidase inhibitor panel for pharmacological target validation. Free Radic. Biol. Med..

[24] Montezano A.C., Burger D., Paravicini T.M., Chignalia A.Z., Yusuf H., Almasri M., He Y., Callera G.E., He G., Krause K.H. (2010). Nicotinamide adenine dinucleotide phosphate reduced oxidase 5 (Nox5) regulation by angiotensin II and endothelin-1 is mediated via calcium/calmodulin-dependent, rac-1-independent pathways in human endothelial cells. Circ. Res..

[25] Goncharov N.V., Avdonin P.V., Nadeev A.D., Zharkikh I.L., Jenkins R.O. (2015). Reactive oxygen species in pathogenesis of atherosclerosis. Curr. Pharm. Des..

[26] Riba A., Deres L., Sumegi B., Toth K., Szabados E., Halmosi R. (2017). Cardioprotective effect of resveratrol in a postinfarction heart failure model. Oxid. Med. Cell Longev..

[27] Ren J., Su D., Li L., Cai H., Zhang M., Zhai J., Li M., Wu X., Hu K. (2019). Anti-inflammatory effects of Aureusidin in LPS-stimulated RAW264.7 macrophages via supressing NF-ᴋB and activating ROS- and MAPKs-dependent Nrf2/HO-1 signaling pathways. Toxicol. Appl. Pharmacol..

[28] Tsai M.H., Hsu L.F., Lee C.W., Chiang Y.C., Lee M.H., How J.M., Wu C.M., Huang C.L., Lee I.T. (2017). Resveratrol inhibits urban particulate matter-induced COX-2/PGE_2_ release in human fibroblast-like synoviocytes via the inhibition of activation of NADPH oxidase/ROS/NF-ᴋB. Int. J. Biochem. Cell Biol..

[29] Su L., Wang Z., Huang F., Lan R., Chen X., Han D., Zhang L., Zhang W., Hong J. (2018). 18β-Glycyrrhetinic acid mitigates radiation-induced skin damage via NADPH oxidase/ROS/p38MAPK and NF-ᴋB pathways. Environ. Toxicol. Pharmacol..

[30] Serrander L., Jaquet V., Bedard K., Plastre O., Hartley O., Arnaudeau S., Demaurex N., Schlegel W., Krause K.H. (2007). NOX5 is expressed at the plasma membrane and generates superoxide in response to protein kinase C activation. Biochimie.

[31] Hussain M., Awan F.R. (2018). Hypertension regulating angiotensin peptides in the pathobiology of cardiovascular disease. Clin. Exp. Hypertens..

[32] Montezano A.C., Nguyen Dinh Cat A., Rios F.J., Touyz R.M. (2014). Angiotensin II and vascular injury. Curr. Hypertens. Rep..

[33] Cheshmehkani A., Senatorov I.S., Dhuguru J., Ghoneim O., Moniri N.H. (2017). Free-fatty acid receptor-4 (FFA4) modulates ROS generation and COX-2 expression via the C-terminal β-arrestin phosphosensor in Raw 264.7 macrophages. Biochem. Pharmacol..

[34] Pérez-Girón J.V., Palacios R., Martín A., Hernanz R., Aguado A., Martínez-Revelles S., Barrús M.T., Salaices M., Alonso M.J. (2014). Pioglitazone reduces angiotensin II-induced COX-2 expression through inhibition of ROS production and ET-1 transcription in vascular cells from spontaneously hypertensive rats. Am. J. Physiol. Heart Circ. Physiol..

[35] Liang X., Yang L.X., Guo R., Shi Y., Hou X., Yang Z., Zhou X., Liu H. (2017). Atorvastatin attenuates plaque vulnerability by downregulation of EMMPRIN expression via COX-2/PGE2 pathway. Exp. Ther. Med..

[36] Gargiulo S., Rossin D., Testa G., Gamba P., Staurenghi E., Biasi F., Poli G., Leonarduzzi G. (2018). Up-regulation of COX-2 and mPGES-1 by 27-hydroxycholesterol and 4-hydroxynonenal: A crucial role in atherosclerotic plaque instability. Free Radic. Biol. Med..

[37] Guzik T.J., Chen W., Gongora M.C., Guzik B., Lob H.E., Mangalat D., Hoch N., Dikalov S., Rudzinski P., Kapelak B. (2008). Calcium-dependent NOX5 nicotinamide adenine dinucleotide phosphate oxidase contributes to vascular oxidative stress in human coronary artery disease. J. Am. Coll. Cardiol..

[38] Cardenas S. (2018). ROS and redox signaling in myocardial ischemia-reperfusion injury and cardioprotection. Free Radic. Biol. Med..

[39] Hahn N.E., Meischl C., Kawahara T., Musters R.J., Verhoef V.M., van der Velden J., Vonk A.B., Paulus W.J., van Rossum A.C., Niessen H.W. (2012). NOX5 expression is increased in intramyocardial blood vessels and cardiomyocytes after acute myocardial infarction in humans. Am. J. Pathol..

[40] Cuccurullo C., Fazia M.L., Mezzetti A., Cipollone F. (2007). COX-2 expression in atherosclerosis: The good, the bad or the ugly?. Curr. Med. Chem..

[41] Liu H., Xu Z., Sun C., Gu D., Teng X., Zhao Y., Zheng Z. (2017). A variant in COX-2 gene is associated with left main coronary artery disease and clinical outcomes of coronary artery bypass grafting. Biomed. Res. Int..

[42] Ross S., Eikelboom J., Anand S.S., Eriksson N., Gerstein H.C., Mehta S., Connolly S.J., Rose L., Ridker P.M., Wallentin L. (2014). Association of cyclooxygenase-2 genetic variant with cardiovascular disease. Eur. Heart J..

[43] Guo Y., Nong Y., Tukaye D.N., Rokosh G., Du J., Zhu X., Book M., Tomlin A., Li Q., Bolli R. (2019). Inducible cardiac-specific overexpression of cyclooxygenase-2 (COX-2) confers resistance to ischemia/reperfusion injury. Basic Res. Cardiol..

[44] Patrono C. (2016). Cardiovascular effects of cyclooxygenase-2 inhibitors: A mechanistic and clinical perspective. Br. J. Clin. Pharmacol..

