# Supplementary material for: Induction of Cyclooxygenase-2 by Overexpression of the Human NADPH Oxidase 5 (NOX5) Gene in Aortic Endothelial Cells
Source: Cells. 2020 Mar 6;9(3):637. doi: 10.3390/cells9030637 (PMC7140418; doi:10.3390/cells9030637)
Supplement: Supplementary file 1 [file cells-09-00637-s001.pdf]

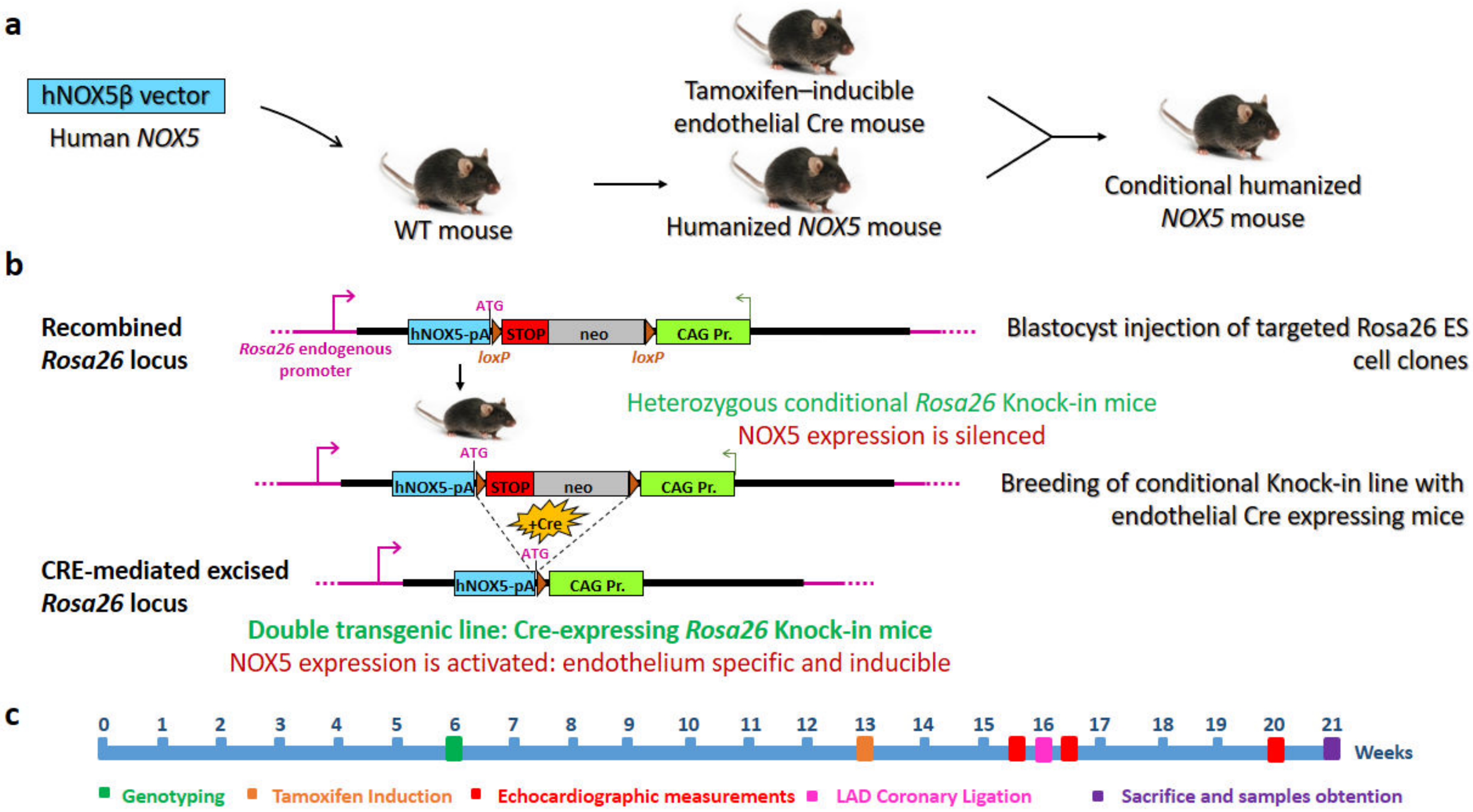

Figure S1. **Generation of the endothelial CRE-expressing knock-in mouse and experimental design.** (**a**) Representative schematic of the humanized NOX5 mouse. NOX5 gene located on the chromosome 6 was engineered in heterozygosis, thus resulting in mice with a single copy of the gene both in males and females. (**b**) Construction of the conditional humanized NOX5-KI mouse. Purple and black lines represent genomic sequences located in the endogenous Rosa26 locus and in the targeting vector to insert NOX5, respectively. LoxP sites are shown as brown triangles. The NOX5 cDNA plus the hGH polyA are depicted as a blue box. The combined STOP-neomycin cassette is represented by a red-grey box and the CAG promoter by a green box. (**c**) Representative schedule of the humanized NOX5 infarcted mice.

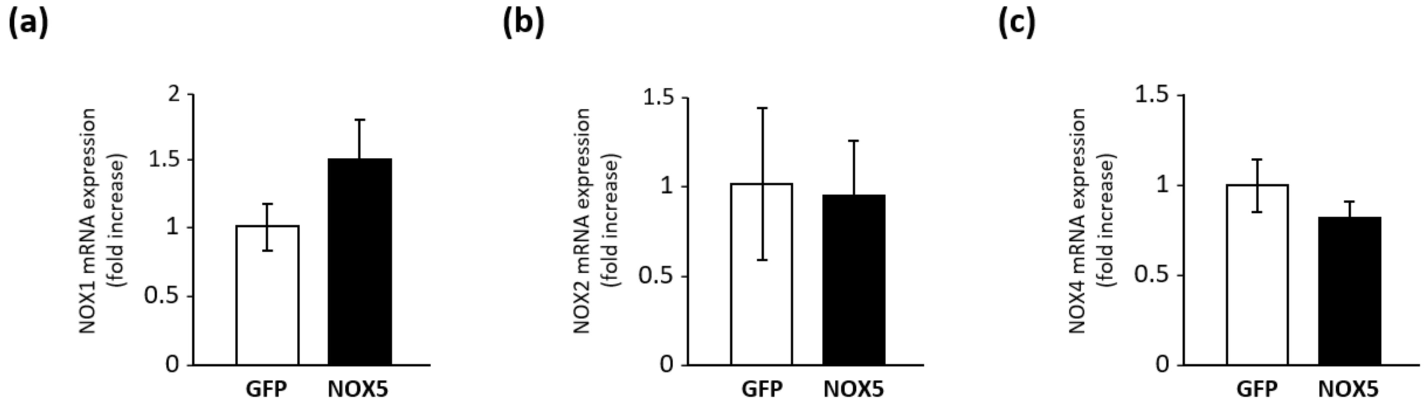

Figure S2. Effects of NOX5-β overexpression on NOX expression in TeloHAEC. (a) NOX1 **(b)** NOX2 and **(c)** NOX4 mRNA levels in NOX5-β and GFP-infected cells for 24 h. n=6.

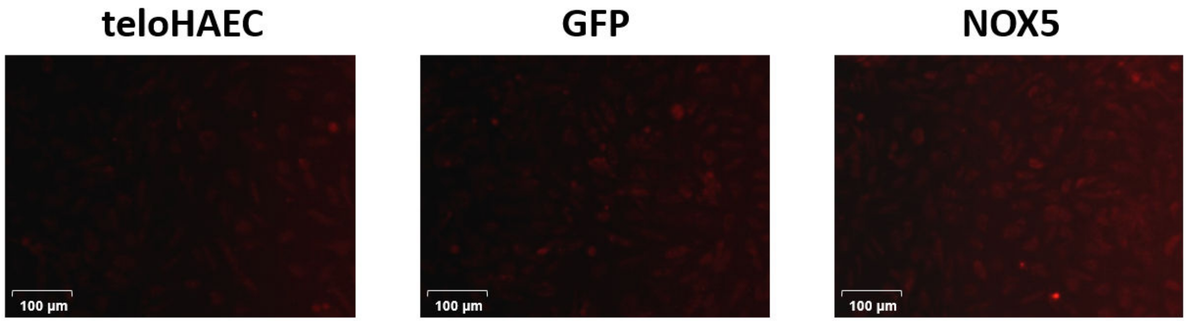

Figure S3. Effect of NOX5-β overexpression on DHE oxidation in TeloHAEC. Images show superoxide production of non-infected cells and cells infected with GFP or NOX5-β adenoviruses. Briefly, TeloHAEC cells were cultured in 96-well black clear-bottom plates and infected with adenovirus encoding GFP or NOX5-β. Next day, cells were washed and incubated at 37 °C in the dark with 100 µM DHE for 5 min. Then, cells were washed and fluorescent images were obtained in a ZOE Fluorescent Cell Imager (Bio-Rad). Representative images are shown.

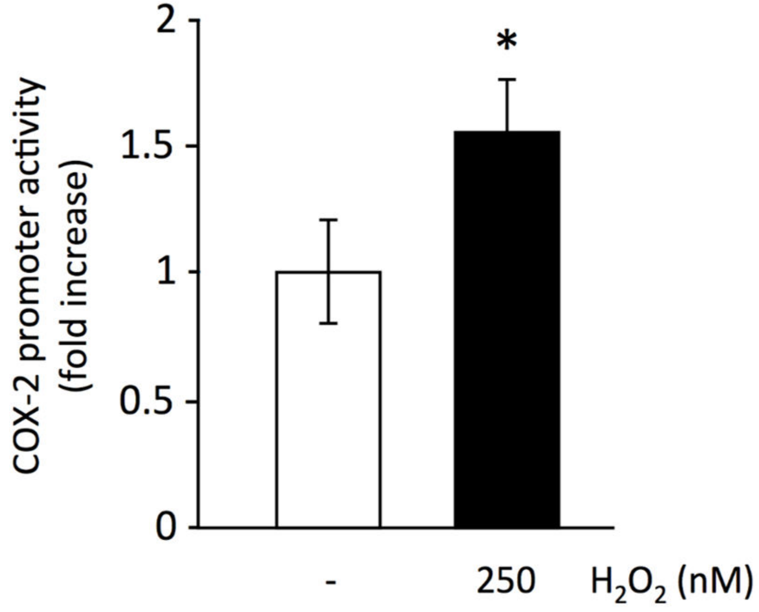

Figure S4. Effect of H_2O_2 on COX-2 promoter transcriptional activity in Telo HAEC. n=6. *p<0.05.

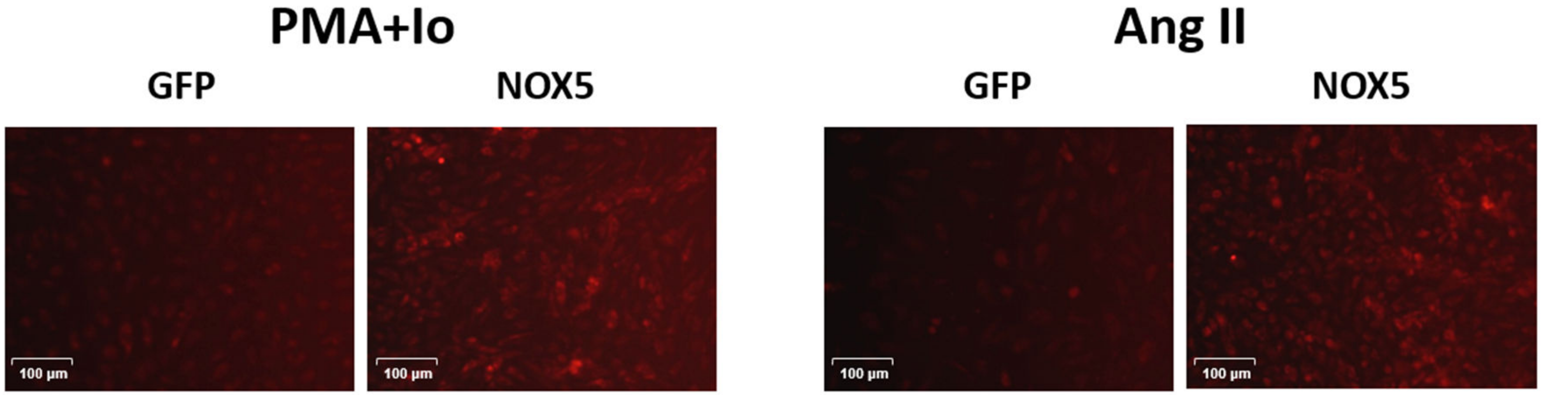

Figure S5. Effect of stimulation with PMA+Io or angiotensin II (Ang II) on superoxide production in GFP- and NOX5-β-infected TeloHAECs. Stimulation with PMA+Io and Ang II enhanced DHE oxidation more in NOX5-β infected cells than in GFP infected cells. Images show superoxide production of cells infected with GFP or NOX5-β adenoviruses, and stimulated with PMA+Io or Ang II. Briefly, TeloHAEC cells were cultured in 96-well black clear-bottom plates and infected with adenovirus encoding GFP or NOX5-β. Next day, cells were stimulated with PMA+Io or with Ang II for 1 h and finally incubated at 37 °C in the dark with 100 µM DHE for 5 min. Then, cells were washed and fluorescent images were obtained in a ZOE Fluorescent Cell Imager (Bio-Rad). Representative images are shown.

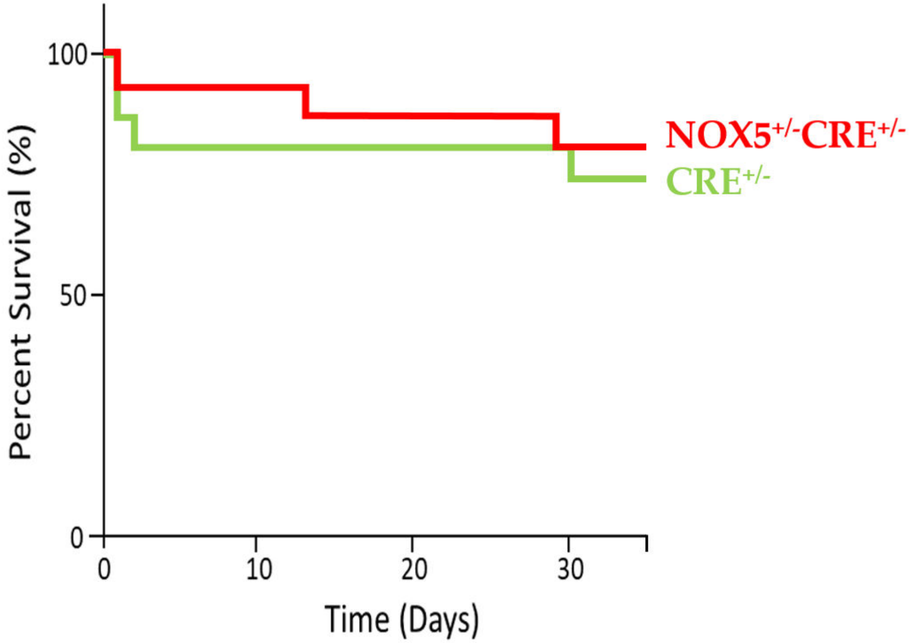

Figure S6. Survival analysis after permanent ligation of the left anterior descending coronary artery (LAD). CRE$^{+/-}$: mice with CRE$^{+/-}$ genotype (n=16). NOX5$^{+/-}$CRE$^{+/-}$: mice with NOX5$^{+/-}$CRE$^{+/-}$ genotype. (n=16).

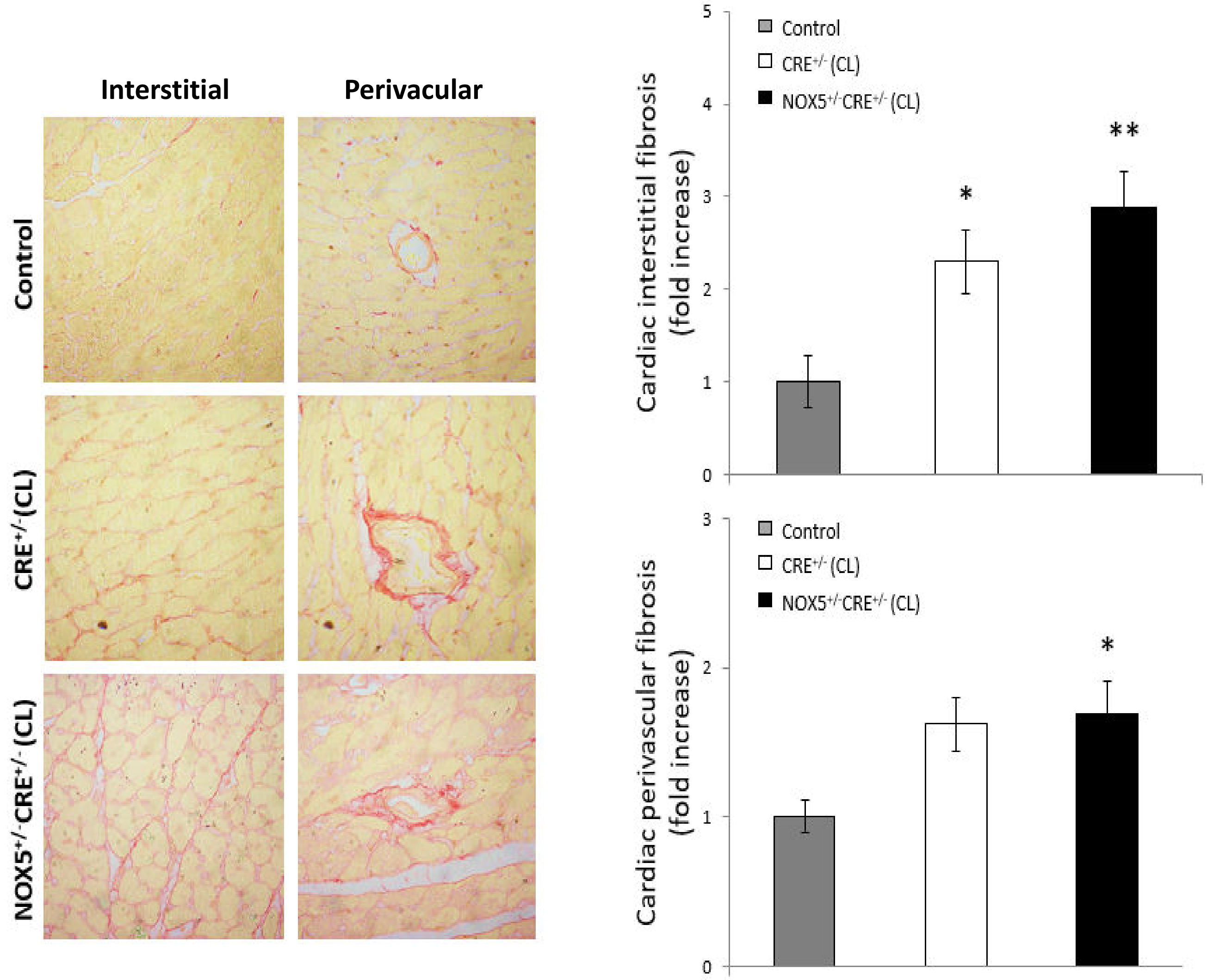

Figure S7. Myocardial interstitial and perivascular fibrosis. Control: mice with no LAD coronary ligation (N=10). $CRE^{+/-}$ (CL): mice with CRE genotype and LAD coronary ligation (N=12). $NOX5^{+/-}CRE^{+/-}$: mice with $NOX5^{+/-}CRE^{+/-}$ genotype and coronary ligation (N=13). *p<0.05 *vs* Control. **p<0.001 *vs* Control. Data are represented as mean±SEM. Histological pictures were taken at 20x. LAD, ligation of the left anterior descending coronary artery. CL, coronary ligation.

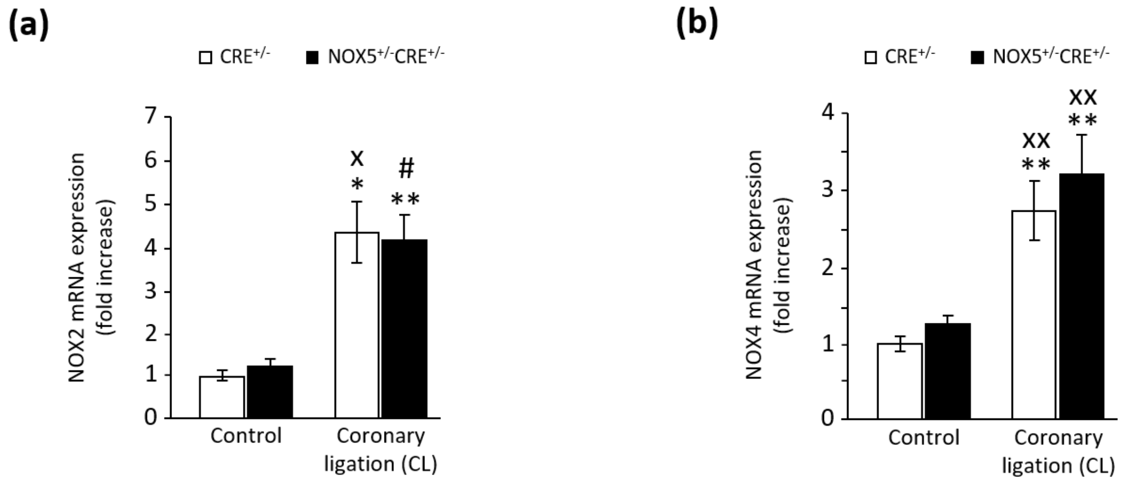

Figure S8. Quantification of NOX enzymes in the heart of mice. (a) NOX2 mRNA expression in control and infarcted mice. $^{*}p<0.05$ *vs* CRE$^{+/-}$, $^{**}p<0.01$ *vs* CRE$^{+/-}$, $^{x}p<0.05$ *vs* NOX5$^{+/-}$CRE$^{+/-}$, $^{xx}p<0.01$ *vs* NOX5$^{+/-}$CRE$^{+/-}$. **(b)** NOX4 mRNA expression in control and infarcted mice. $^{**}p<0.01$ *vs* CRE$^{+/-}$, $^{xx}p<0.01$ *vs* NOX5$^{+/-}$CRE$^{+/-}$. Control, control mice with no MI. Coronary ligation (CL), mice that suffered MI by LAD coronary ligation. CRE$^{+/-}$, mice with CRE$^{+/-}$ genotype (n=9 for CRE$^{+/-}$ and NOX5$^{+/-}$CRE$^{+/-}$ groups). NOX5$^{+/-}$CRE$^{+/-}$, mice with NOX5$^{+/-}$CRE$^{+/-}$ genotype (n=12 for CRE$^{+/-}$CL group, and n=13 for NOX5$^{+/-}$CRE$^{+/-}$CL group).

NOX5 mRNA was detected only in NOX5$^{+/-}$CRE$^{+/-}$ mice, although no differences were found between control and MI groups (*data not shown*).
